# Chemical Exposures: Exploring Developmental Origins of Obesity

**DOI:** 10.1289/ehp.115-a242

**Published:** 2007-05

**Authors:** Graeme Stemp-Morlock

In the past 20 years, obesity rates across the developed world have skyrocketed. About one-third of U.S. adults are overweight and another third are obese, according to the National Institute of Diabetes and Digestive and Kidney Diseases. Similarly, more than one-third of U.S. children are overweight or at risk for overweight. The result is a host of health problems for millions of people, including diabetes, coronary heart disease, hypertension, hypercholesterolemia, gall-bladder disease, and pregnancy complications. Researchers have typically blamed two main factors: reduced physical activity and increased caloric intake. But perhaps that’s not all there is to it. In a session at the 2007 annual meeting of the American Association for the Advancement of Science titled “Obesity: Developmental Origins and Environmental Influences,” scientists looked at the question of whether prenatal chemical exposure may be predisposing some children to a life of obesity.

During the session, Frederick vom Saal, a professor of biological sciences at the University of Missouri–Columbia, described a link between bisphenol A and obesity. Bisphenol A is a major component of polycarbonate plastics found in the linings of cans and in baby bottles. It can leach from plastic when it is heated or there is a change in acid–base balance.

According to vom Saal, bisphenol A has exhibited endocrine disruption in animals and humans at ppb doses. “There are situations where bisphenol A is causing effects at a thousand times lower than the amount in the average human body,” he said, adding that current global production of bisphenol A runs about 7 billion pounds per year.

In one of vom Saal’s experiments, presented at the meeting, pregnant mice were fed doses of bisphenol A up to 10 times lower than what finds its way into the average human. Although the offspring did not meet the criteria for obesity, they did exhibit abnormal growth later in life. Other studies have linked similar doses with cancer of the prostate and mammary gland in offspring.

Retha Newbold, a biologist with the Developmental Endocrinology Studies Group of the NIEHS Laboratory of Molecular Toxicology, focused her attention on diethylstilbestrol (DES), a potent estrogen widely prescribed from the 1940s to the 1970s for pregnancies at risk of miscarriage. The compound has been shown to cause impaired fertility, reproductive tract malformations, and a low incidence of cancer in the children of women who took the drug in pregnancy.

Newbold and her colleagues performed an experiment in which mice were exposed to DES in the womb or shortly after birth. DES-exposed mice were smaller than untreated controls after birth. However, when the animals reached puberty, the DES-treated mice became significantly larger than the controls. Activity levels and food consumption were similar for obese DES-treated mice and normal-weight controls. Yet DES mice accumulated more body fat and in some cases a more difficult time processing glucose.

In a third presentation during the session, Bruce Blumberg, an associate professor with the University of California, Irvine, Department of Developmental and Cell Biology, discussed how tributyltin alters gene expression to promote fat cell differentiation. Tributyltin is an organotin used as a heat stabilizer in the manufacture of polyvinyl chloride plastics that, like bisphenol A, may leach out of the plastic.

Testing of pure tributyltin showed that the compound altered receptor sensitivity at very high potency, and at similar levels to drugs that specifically target that receptor. “Prenatal tributyltin exposure causes permanent physiological changes in these animals that predisposes them to gaining weight,” said Blumberg. “They were not treated with any more tributyltin after that prenatal exposure, they had a normal diet, normal exercise, and yet they were significantly fatter.”

## Figures and Tables

**Figure:**
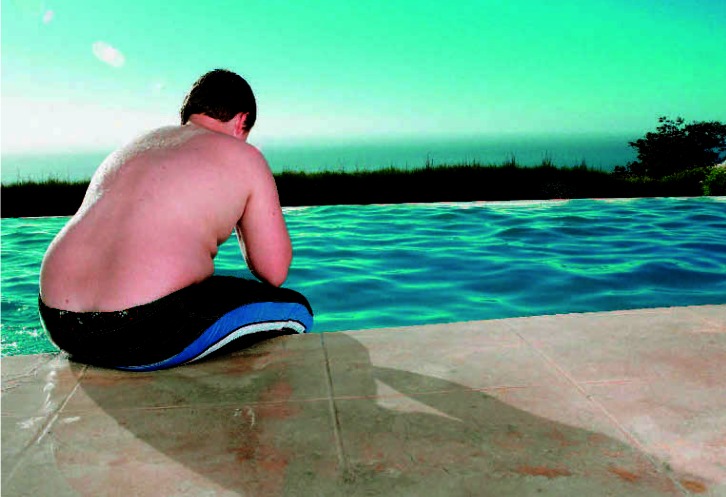
A new line of thinking Evidence now suggests that a myriad of prenatal chemical exposures may predispose children to obesity later in life.

